# Supplementary material for: Frailty, Health Literacy, and Self-Care in Patients with Chronic Kidney Disease in Taiwan
Source: Int J Environ Res Public Health. 2022 Apr 28;19(9):5350. doi: 10.3390/ijerph19095350 (PMC9102108; doi:10.3390/ijerph19095350)
Supplement: Supplementary file 1 [file ijerph-19-05350-s001.zip › ijerph-1617010-supplementary.pdf]

Appendix: Supplementary data

Chronic kidney disease (CKD) self-care scores by item among studied CKD patients (N = 144)

	Mean ± SD
**Medication adherence**	**4.8 ± 0.6**
1. I myself may change the prescribed dosing time	4.7 ± 0.7
2. I myself may stop taking drugs	4.8 ± 0.5
3. I may not keep taking drugs continuously	4.8 ± 0.5
4. I myself may change prescribed dosing frequency	4.8 ± 0.7
5. I myself may change prescribed drug dosage	4.9 ± 0.5
**Diet control**	**3.5 ± 1.1**
1. I always follow the principle of kidney disease diet when eating a meal	2.7 ± 1.0
2. I always eat adequate amount of food based on health professionals' Recommendations	3.6 ± 1.2
3. I always control food and nutrition ingestion	4.2 ± 1.0
4. I always follow principle of kidney disease diet even in holidays or dining out Exercise	3.4 ± 1.2
**Exercise**	**3.2 ± 1.4**
1. I always keep regular exercise	3.5 ± 1.4
2. I may still try to work out for keeping my kidney disease under control whenever I do not want to do exercise	3.4 ± 1.4
3. I may still try to take time out of my busy schedule to work out	2.8 ± 1.5
**Smoking behaviour**	**4.2 ± 1.6**
1. I do not smoke every day	4.4 ± 1.5
2. Anyone around smoking I will stop or avoid	4.0 ± 1.6
**Blood pressure monitoring**	**3.1 ± 1.6**
1. I always monitor my blood pressure	2.9 ± 1.6
2. I may increase frequency of monitoring my blood pressure when not feeling well	3.3 ± 1.6

Chronic kidney disease (CKD) health literacy scores by item among studied CKD patients (N = 144)

	Answer True Number (N)	Answer True (%)
**Functional health literacy**		
1. As shown in the above table, if the glomerular filtration rate (eGFR) index is 20 ml/min/1.73m2,what stage of the chronic kidney disease is?	103	71.5%
2. If the daily calories intake are 1500 kcal, how many servings of beans, fish, meat, and eggs do you need to consume?	93	64.6%
3. How many grams of protein per 100 grams in the nutrition label shown above?	105	72.9%
4. What should Mr. Hao avoid to consume concomitantly while taking the medicine mentioned in the medicine bag?	100	69.4%
5. Which one of side effects may not be presented after Mr. Hao taking this medicine?	95	66%
**Communicative health literacy**		
1. Nurse: "What stage of urination should you collect urine for the urinalysis?	79	54.9%
2. What is suggested to avoid by doctors for fear of nocturnal urinary frequency?	85	59%
3. What should you do after receiving the computed tomography examination with the contrast agent?	90	62.5%
4. The doctor suggested: " the patient with chronic kidney disease has to limit the amount of water intake if suffering from serious edema. The amount of daily water intake is the amount of urine output of previous day added with 500-700 C.C. What is the appropriate amount of water intake today if the amount of urine output was 700 c.c. yesterday?	59	40.9%
5. What time should Mr. Hao take the next dose if he took the medication after the breakfast in this morning?	118	81.9%
6. Which one is the antihypertensive agent for Mr. Hao's hypertension?	133	92.4%
7. What is your suggestion if Mr. Hao suffering from declined urine output recently, lower limbs swelling sensation while walking, poor appetite, and vomiting after eating congee for lunch?	124	86.1%
**Critical health literacy**		
1. What would you do if a friend sent you the following message through a communication software?	108	75%
2. How many bowls of carbohydrate food can you eat for dinner if you have had one bowl of carbohydrate food and two servings of meat today?	78	54.2%
3. How many grams of protein do you intake if you eat 200 grams of biscuits?	94	65.3
4. How many units of erythropoietin in total in a week should Mr. Hao be	103	71.5%

	administered?		
5.	Mr. Hao bought 24 ampoules of erythropoietin on January 1, 2017, and still have not run out. Can the remaining erythropoietin be administered if his laboratory examination of erythrocytes count was low on January 1, 2018?	27	88.2%
